# Prevalence and control of hypertension in a high HIV-prevalence setting, insights from a population based study in Botswana

**DOI:** 10.1038/s41598-023-44499-4

**Published:** 2023-10-19

**Authors:** Mosepele Mosepele, Kara Bennett, Tendani Gaolathe, Joseph M. Makhema, Mompati Mmalane, Molly Pretorius Holme, Refeletswe Lebelonyane, Omolola Ometoruwa, Lisa A. Mills, Kathleen M. Powis, Jean Leidner, Joseph N. Jarvis, Neo M. Tapela, Tiny Masupe, Lucky Mokgatlhe, Virginia A. Triant, Kathleen E. Wirth, Thato Moshomo, Shahin Lockman

**Affiliations:** 1 Department of Internal Medicine, Faculty of Medicine, University of Botswana, Gaborone, Botswana; 2 Botswana-Harvard AIDS Institute Partnership, Gaborone, Botswana; 3 Department of Immunology & Infectious Diseases, Harvard T. H. Chan School of Public Health, Boston, MA USA; 4 Bennett Statistical Consulting Inc, Ballston Lake, New York, USA; 5 Ministry of Health & Wellness, Gaborone, Botswana; 6 Division of Infectious Diseases, Brigham & Women’s Hospital, Boston, MA USA; 7 Centers for Disease Control and Prevention (CDC), Atlanta, USA; 8 Department of Medicine, Massachusetts General Hospital, Boston, MA USA; 9 Goodtables Data Consulting, Norman, OK USA; 10 London School of Hygiene and Tropical Medicine, London, United Kingdom; 11 Division of Global Health Equity, Brigham and Women’s Hospital, Boston, MA USA; 12 Department of Family Medicine & Public Health, Faculty of Medicine, University of Botswana, Gaborone, Botswana; 13 Department of Biostatistics, Faculty of Social Sciences, University of Botswana, Gaborone, Botswana; 14 Department of Epidemiology, Harvard T. H. Chan School of Public Health, Boston, MA USA; 15 Sir Ketumile Masire Teaching Hospital, Department of Internal Medicine, Faculty of Medicine, University of Botswana, 3rd Floor, Block F, Room F4069, Gaborone, Botswana

**Keywords:** Population screening, HIV infections, Hypertension

## Abstract

In a population-based representative sample of adults residing in 22 communities in Botswana, a southern African country with high HIV prevalence, 1 in 4 individuals had high blood pressure. High blood pressure was less prevalent in adults with HIV than without HIV. Sixty percent of persons with high blood pressure had not previously been diagnosed. Among individuals with a prior diagnosis of high blood pressure who reported being prescribed anti-hypertension medications, almost half had elevated blood pressure, irrespective of HIV-status. One-third of adults in this setting (mainly men) declined free non-invasive blood pressure assessments in their households. In conclusion, our study highlights alarmingly high hypertension rates in the community, with low levels of awareness and control, emphasizing the urgent need for community level BP screening and active management to reach recommended targets.

## Introduction

Globally, the prevalence of hypertension increased by approximately 64% and 35% between 1997–2007 and 2007–2017 periods respectively and was linked to an estimated 10.4 million deaths worldwide in 2017^1^. Currently, hypertension affects almost a third of the global population, of whom an estimated 40% are not aware of their high blood pressure (BP), and only a third of those with a known diagnosis of hypertension have attained the recommended BP target of <140mmHg systolic and <90mmHg diastolic^2^. The Africa region had both the lowest prevalence of diagnosed hypertension (estimated 1 in 4 adults) and the lowest rates of BP control among those with a known diagnosis of hypertension (estimated at 15%)^2^. Sub-Saharan Africa is also home to approximately two-thirds of people living with human immunodeficiency virus (PLHIV)^3^. These estimated 25 million PLHIV are at increased risk for cardiovascular disease^4^, and data from Sub-Saharan Africa show high rates of cardiovascular disease^5,6^. Data are conflicting as to whether PLHIV are more likely to develop hypertension (particularly after initiation of antiretroviral treatment [ART]) than HIV-negative persons, and most data on the topic are from North America and Europe^7,8^. The prevalence of hypertension in PLHIV versus in the general population, and the extent to which blood pressure targets are met among PLHIV who are diagnosed with hypertension, are not well-understood in Sub-Saharan African countries (including in Botswana, where more than 80% of all PLHIV are on ART and in longitudinal care)^9^.

We had a unique opportunity within the population-based Ya Tsie HIV prevention trial (performed in communities across Botswana) to evaluate community prevalence of high BP and then assess whether PLHIV had a higher prevalence of high BP. We also evaluated the control of blood pressure (overall and by HIV status) among individuals with an established diagnosis of hypertension who were already on antihypertensive medications.

## Methods

### Study design

We performed a one-time BP assessment between March 2017 and June 2018 via home-based assessments, nested in the cluster-randomized Ya Tsie HIV prevention trial (the Botswana Combination Prevention Project [BCPP]), which was conducted in 30 rural and peri-urban communities in Botswana (combined population ~180,000, which is ~10% of Botswana’s population)^10,11^. The trial was designed to assess the efficacy of a combination of enhanced HIV diagnosis, linkage, treatment, and prevention interventions in reducing HIV incidence over a 30-month period.

### Setting and participants

As part of Ya Tsie, we recruited a representative, population-based longitudinal cohort of all consenting 18-64-year old adults residing in a random sample of 20% of households in each of the 30 communities^10,11^. During the third and final household survey for the main Ya Tsie trial, all adults above 18 years of age (irrespective of pregnancy statuses) were invited to participate in the blood pressure assessment sub-study. Only 22 out of 30 communities participated in the blood pressure assessment because 8 communities had completed Ya Tsie trial participation by the time that regulatory approvals were obtained for the blood pressure assessment.

### Study procedures

Trained research assistants administered a questionnaire to establish any prior/current diagnosis of hypertension or use of antihypertensive medications. Research assistants also reviewed participants’ outpatient medical cards for evidence of prior hypertension diagnosis and treatment. Detailed sociodemographic and health data (including HIV and ART status and HIV-1 RNA) were collected in all Ya Tsie participants as detailed in the main study publications^11^. Bilateral upper arm BP measurements in duplicate were obtained in the home setting following standard guidelines for BP measurement^12^ (using Healthease© automated BP machines with different cuff sizes available, to ensure that the cuff covered 80% or more of the participant’s upper arm). Thus, a total of four BP measurements were obtained for each participant. Waist and hip circumferences were also measured twice in each participant using a non-stretchable tape measure under the participant’s clothes with the participant in the upright erect position^13^. Waist circumference was measured at the midpoint (level of umbilicus). Hip circumference was measured at the widest hip circumference (most protuberant buttock and lateral upper thigh).

### Statistical considerations

High BP was defined as having either: (1) a pre-study diagnosis of hypertension (by self-report or by medical record indication of diagnosis of hypertension or use of antihypertensive medications at the time of participation in the hypertension sub-study), or (2) raised BP measurement at the time of the study visit (defined as either mean systolic BP ≥140mmHg and/or mean diastolic BP ≥90mmHg, from the bilateral duplicate measurements). Baseline continuous characteristics were compared using the Wilcoxon test (for continuous variables) or the chi-square test (for categorical variables).

Prevalence and prevalence ratios (PR) of the binary outcome of high BP were derived from modified Poisson generalized estimating equations, along with corresponding Huber robust standard estimates (SEs) and 95% Wald confidence intervals (CIs)^14^; association with HIV status was examined in these models. A similar analysis assessed for the binary outcome of controlled blood pressure (two pre -set BP cut-off points: BP <140/90 mmHg, and then BP <130/80 mmHg) among those taking hypertension medication at the time of enrollment. We adjusted for missing BP measurements (see below), and age/sex in the fully adjusted prevalence ratios (aPRs).

Of those taking part in this hypertension sub-study, 1198 (30%) had missing BP readings. To account for potential selection bias due to missing outcome data, we applied complete-case inverse probability weighting (IPW) to re-estimate the overall prevalence of high BP and the corresponding prevalence ratios. Our approach adjusts for missing blood pressure readings by accounting for potential correlates between missing blood pressure reading and other baseline demographic, clinical, and social characteristics^15^. To build the multivariate logistic regression required for the IPW adjustment approach, we used a stepwise selection procedure (entry and exit criteria set to p<0.2) to identify covariates of known or hypothesized risk factors for high BP as listed in detail in Supplementary Table 1. We included missing indicators for each selected variable in the final logistic regression model for the weights to maximize the number of cases included in the final model and to maintain a constant sample size across analyses. The final model applied this IPW method and adjusted for age and sex.

BP control was assessed among participants who had a previous diagnosis of hypertension and who were prescribed anti-hypertension medications. BP control was defined as having both systolic BP <140 mmHg and diastolic BP <90 mmHg. Assessment of adjusted prevalence of BP control followed the same strategy as model estimates for high BP prevalence. BP control was further assessed in sensitivity analysis using the 2017 AHA/ACC alternative reasonable targets for BP control defined as blood pressure <130/80 mmHg (used when BP is measured in the household/community setting)^16^. All analyses were performed using SAS Version 9.4 (SAS Institute, Cary, NC, USA). Results with p-values <0.05 were considered significant in all analyses unless stated otherwise.

### Ethical considerations

Written informed consent was obtained from all the study participants. Consent was performed in either Setswana or English. When dealing with respondents who were illiterate and unable to provide written informed consent, we employed an oral informed consent procedure, which involved verbal explanation and consent, in the presence of a witness, and a thumbprint as a signature.

All study materials and procedures were approved by the Botswana Ministry of Health and Wellness Human Research Development Committee (HRDC) and the United States Centers for Disease Control and Prevention (CDC) Institutional Review Board. All methods and procedures were caried out in compliance with relevant guidelines and regulations.

### Conference presentation

Parts of the analysis and results of this work were presented at Conference on Retroviruses and Opportunistic Infections (CROI), March 2018, Boston, MA, USA, by Mosepele Mosepele.

## Results

### Baseline demographics

Among 3981 participants approached for BP assessment, 2783 (70%) agreed to have BP measured while 1198 (30%) did not agree to have BP measured (Table 1 below).

**Table 1:** Baseline demographics and clinical characteristics of participants, Ya Tsie BP Assessment, Botswana, 2017-2018.

Characteristics	Available BP				BP missing	
	All	PLHIV	HIV-negative	*P-value	All, N=1198	**P-value
	N=2783	N=794	N=1980			
Age (years), median (IQR)	35.8 (26.8, 49)	42.2 (35, 50.8)	32.5 (25, 47.5)	<0.01	36.3 (26.9, 47.7)	0.67
17–24, N (%)	526 (19)	31 (4)	489 (25)	<0.01	229 (19)	0.26
25–34, N (%)	796 (29)	162 (20)	633 (32)		322 (27)	
35–44, N (%)	594 (21)	279 (35)	315 (16)		284 (24)	
45–54, N (%)	410 (15)	195 (25)	215 (11)		188 (16)	
55–68, N (%)	457 (16)	127 (16)	328 (17)		175 (15)	
Female, N (%)	1842 (66)	600 (76)	1234 (62)	<0.01	705 (59)	<0.01
Education level						
Non-formal, N (%)	324 (12)	112 (14)	212 (11)	<0.01	143(12)	0.52
Primary, N (%)	582 (21)	250 (32)	330 (17)		254(21)	
Secondary, N (%)	1469 (53)	387 (49)	1078 (55)		606 (51)	
Higher than secondary, N (%)	396 (14)	40 (5)	353 (18)		189(16)	
Waist-hip ratio*^*						
Females, median (IQR)	0.84 (0.79, 0.91)	0.85 (0.80, 0.92)	0.84 (0.78, 0.91)	<0.01	0.85 (0.76, 0.93)	^
Males, median (IQR)	0.87 (0.81, 0.93)	0.88 (0.83, 0.94)	0.86 (0.81, 0.93)	0.01	0.89 (0.85, 0.97)	^
Cigarette smoking						
Ever, N (%)	363 (13)	113 (14)	250 (13)	0.26	171 (14)	0.3
Current, N (%)	234 (8)	65 (8)	169 (9)	0.77	123 (10)	0.06
Blood pressure, mmHg						
Systolic, median (IQR)	119 (109, 130)	117 (108, 128)	119 (109, 130)	0.01	N/A	N/A
Diastolic, median (IQR)	80 (72, 88)	80 (72, 88)	80 (72, 88)	0.66	N/A	N/A
Current antihypertensive medication						
N (%)	276 (10)	84 (11)	192 (10)	0.48	74 (6)	<0.01
CD4 count, cells/ul, median (IQR)	N/A	554 (417, 748)	N/A	N/A	534 (381, 678)	0.37
ART status						
On ART, N (%)	N/A	749 (94)	N/A	N/A	361 (90)	0.01
ART-naïve, N (%)	N/A	34 (4)	N/A		36 (9)	
ART defaulter, N (%)	N/A	11 (1)	N/A		5 (1)	

All values are count (N) and associated percentages, mean (SD) or median (IQR).

*PLHIV* people living with HIV, *IQR* inter quartile range, *HIV* human immune deficiency virus, *ART* antiretroviral therapy, *N/A* not applicable.

CD4 count; most recent/last CD4 count available in the medical records.

*Comparisons made between PLWH and those who are HIV-negative.

**Comparison made between those who agree (N=2783) to have blood pressure measured versus those who refused (N=1198).

^1358 (74%) of the 1842 females with BP agreed to waist-hip measurements. Therefore p-value is not provided since only n=6 women who refused BP but agreed to waist-hip measurement. Also, 670 (71%) of the 942 males with BP agreed to waist-up measurements. Similarly, since only n=11 men who refused BP agreed to waist-hip p-value is not provided.

Age did not significantly differ between those participants who agreed versus who refused to have BP measured (median 36 years in both groups, p = 0.26). However, being female, on anti-hypertensive medication, and on ART were each associated with being more likely to agree to have BP measured (p < 0.01 in all three instances, Table 1) in unadjusted analysis. Supplementary Table 1 summarizes the model selected for IPW, provides additional baseline demographic and clinical characteristics, and compares participants with and without BP readings.

The following results are based upon the 2783 participants with available BP measurements, 29% of whom were PLHIV. PLHIV were approximately 10 years older than participants without HIV, more likely to be female, had fewer years of education, and had higher waist-hip ratios (Table 1). However, rates of cigarette smoking or use of anti-hypertensive medications did not significantly differ by HIV status. Nearly all (94%) PLHIV in this cohort were on ART, and the median CD4 count in PLHIV was high (554 cells/μL).

### Factors associated with refusal of a one-time blood pressure measurement

The most common reasons for refusing BP measurement were not having time (69%) and not being ready to have the measurement performed (15%). In the adjusted model that evaluated predictors of non-participation in the blood pressure measurement (Supplementary Table 2), both PLHIV or persons with unknown HIV-status were less likely to agree to a BP measurement (adjusted odd ratio [aOR] 0.75, 95% CI 0.64–0.88), and aOR 0.30 (95% CI 0.12–0.72), respectively. Similarly, PLHIV on ART were also less likely to agree to BP measurement compared with PLHIV who were not on ART, aOR 0.62 (95% CI 0.39–0.982). Participants who reported any prior use of anti-hypertension medications were significantly more likely to agree to a BP measurement, aOR 2.39 (95% CI 1.13–5.05).

### Hypertension prevalence and control

**Figure 1:**
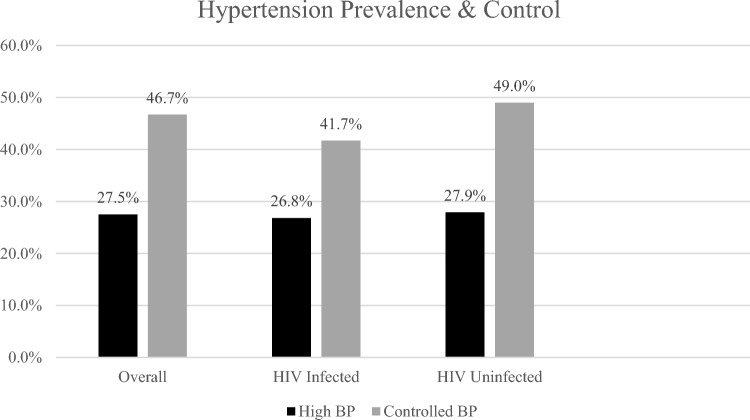
Bar chart showing hypertension prevalence & control overall and by HIV status.

Among the 2783 participants enrolled with measured BP, the overall prevalence of high BP (either pre-existing or new, based upon measured BP) was 27.5% (95% CI 25.1–30.2%) with PLHIV having significantly lower prevalence of high BP, aPR 0.69 (95% CI 0.62–0.76, Table 2 and Fig. 1 below).

**Table 2:** Hypertension prevalence among all, and hypertension control among those on anti-hypertensive treatment, Ya Tsie BP assessment, Botswana, 2017-2018.

	Prevalence of high BP (pre-existing or measured)		Prevalence of Controlled BP among persons reporting current anti-hypertensive use* (<140/90 mmHg)		Prevalence of Controlled BP among persons reporting current anti-hypertensive use** (<130/80 mmHg)	
	N	P (95% CI)	N	P (95% CI)	N	P (95% CI)
Crude prevalence						
Overall	2783	27.5 (25.1, 30.2)	276	46.7 (39.6, 55.2)	276	13.4 (9.1, 19.8)
HIV-infected	794	26.8 (23.3, 30.9)	84	41.7 (30.3, 54.0)	84	10.7 (5.2, 20.7)
HIV-uninfected	1980	27.9 (25.5, 30.5)	192	49.0 (38.7, 59.3)	192	14.6 (9.9, 20.8)
Prevalence adjusted for selection bias^a^						
Overall	2660	26.1 (23.6, 28.8)	263	46.5 (40.1, 54.0)	263	12.6 (7.7, 20.6)
Prevalence ratio (ref: HIV-uninfected)	*N*	*PR (95% CI)*	*N*	*PR (95% CI)*	*N*	*PR (95% CI)*
Crude	2774	0.96 (0.85, 1.09)	N/A	N/A	N/A	N/A
PR adjusted for selection bias^a^	2655	0.98 (0.86, 1.12)	263	0.85 (0.59, 1.22)	263	0.94 (0.55, 1.61)
aPR^b^ adjusted for selection bias^a^	2655	**0.69 (0.62, 0.76)**	263	0.88 (0.60, 1.28)	263	0.94 (0.54, 1.63)

^a^The inverse-probability weighted (IPW) multivariate logistic regression accounted for lack of blood pressure measurement and control for the following co-variates: age, gender, and all covariates from Supplementary Table 2.

^b^In a model adjusted for missing BP and for the interaction between age and gender (since age is categorical with 5 categories, there are 10 categories of age and gender).

*Blood pressure control was defined as BP <140/90 mmHg.

**In sensitivity analysis among participants currently receiving anti-hypertension treatment, BP control was defined as BP <130/80 mmHg and the final fully adjusted mode for missing BP and for age and gender, in this case age is included as continuous and interaction between age and gender is not included as there were not enough participants classified as having controlled BP under the sensitivity definition to support adjustment for categorical variables (n=37 with controlled BP under this sensitivity definition compared to 129 with controlled BP using the cut-off point of <140/90 mmHg). Significant values are bold.

Of the 766 participants with high BP, 307 (40%) had a pre-existing diagnosis of hypertension (while 60% had newly-diagnosed high BP based upon our study BP measurements). Of these 307 participants with known hypertension, 276 (90%) reported currently using anti-hypertensive medications and the remaining 31 (10%) were not taking anti-hypertensive medications (20 or 65% of these 31 individuals had high blood pressure at the time that BP was measured in this study). Living with HIV was not significantly associated with increased prevalence of newly-diagnosed high BP (aPR 0.96, 95% CI 0.80–1.16) nor of being prescribed anti-hypertensive medications (aPR 1.09, 95% CI 0.76–1.56) compared with being HIV-negative.

As noted above, 276 (10%) participants reported active use of anti-hypertensive medications at the time of the survey; of whom 84 (11%) were PLHIV while 192 (10%) were HIV-negative participants. Table 2 shows the proportions of participants who met BP targets of <140/90 mmHg and <130/80 mmHg in persons reporting active use of anti-hypertensive medications. The proportions of treated participants meeting blood pressure targets did not differ in PLHIV versus without HIV: 42% versus 49% for BP target <140/90 mmHg and 11% versus 15% for BP target <130/80 mmHg, respectively (Table 2 below).

## Discussion

In this population-based, representative sample of residents from 22 communities throughout Botswana, one in four relatively young adults (median age 36 years) had high BP. Furthermore, more than half of the participants did not know that they had elevated blood pressure, and almost half of those who reported taking anti-hypertensive medications had BP measurements that exceeded targets. While living with HIV was associated with a lower prevalence of high BP when compared to HIV-negative status, PLHIV were no more likely to have already been diagnosed with or be on treatment for hypertension than their HIV-negative counterparts. 

We found a higher prevalence of high BP (26%) compared with the high BP prevalence observed in a 2007 national survey of ~4000 Botswana adults (also about two-thirds female) between 25 and 64 years (17%)^17^. This may be because the 2007 survey based high BP prevalence only on medical history (known BP assessment by a healthcare worker within 12 months prior) and did not include actual BP evaluation. Given known high rates of undiagnosed high BP in the region (40–60% based on our study and others^2,18–20^), the 2007 Botswana hypertension prevalence survey may have underestimated hypertension prevalence. Our observed overall high BP prevalence of 26% in 2017–2018 is similar to the sub-Saharan Africa prevalence estimates of 25% generated in 2018^2^.

Almost 60% of our study participants with high BP had not previously been diagnosed as having high BP. This is similar to the prevalence of newly-diagnosed high BP (56–59%) reported among adults in low and middle income (LMIC) countries 2003–2009^18^, but higher than the 40% prevalence of undiagnosed hypertension reported among adults in sub-Saharan Africa in 2018^2^. This finding highlights the importance of implementing more effective screening for hypertension in the region.

Living with HIV was associated with lower high BP prevalence, aPR 0.69 (95% CI 0.62–0.76). This association has been observed by others. For instance, in neighboring South Africa, hypertension prevalence was lower among PLHIV versus those without: 19.5% and 27.9%, p<0.001, respectively^21^. Similarly, in the Copenhagen Co-morbidity in HIV Infection (COCOMO) Study of 1099 PLHIV compared with 12,161 age- and sex-matched HIV-uninfected controls, the study reported a decreased risk of having hypertension among PLHIV, aOR 0.63 (95% CI 0.54–0.74). One potential explanation of this difference is that PLHIV are engaged in primary preventative care more often than their HIV-negative counterparts. As a result, PLHIV may be more likely to receive counselling on low salt intake, regular exercise, weight control, and cutting down on alcohol consumption among others, particularly if they are found to have elevated BP measurements during routine HIV care visits^16^. Our results are corroborated by the longitudinal Health and Aging in Africa study of an INDEPTH Community in South Africa (HAALSI) in 5,059 participants over 40 years of age, which revealed that being on ART was associated with increased awareness of hypertension and hypertension treatment^22^. Some studies have shown that PLHIV may have increased risk of hypertension after starting ART compared to those who are ART-naïve^23^, while other studies have not reported any association between ART exposure and risk of hypertension in sub-Saharan Africa^24,25^. We could not evaluate the role of ART initiation in the development of hypertension due to the cross-sectional nature of our study (in addition, nearly all PLHIV taking part in our study were on ART).

The main goal for prescribing anti-hypertensive medications is to attain BP targets^26,27^ and limit end-organ dysfunction associated with chronically elevated BP^16,28,29^. Unfortunately, at best, only one in two participants on anti-hypertensive medication in our study were being adequately treated to achieve BP targets in our study, similar to the reported 45% and 60% BP control among those on anti-hypertensive medications during the 2018 hypertension assessments in sub-Saharan Africa and globally, respectively^2^. Only one in ten participants on anti-hypertensives had attained the more stringent BP cut-off point of <130/80 mmHg. We had expected to detect higher rates of BP control among PLHIV (due to longitudinal engagement in care)^10^. However, we did not observe a significant difference in BP control and prescription of anti-hypertensive medications by HIV group. Patients receiving care in clinics where HIV/blood pressure treatment are integrated have been reported to have higher rates of hypertension diagnosis and control in some^22,30^ but not all^31^ studies. Unfortunately, we could not assess the extent to which our participants received integrated services or not.

Our study had several limitations. Almost 30% of individuals declined BP measurement. Our IPW analysis attempted to address this limitation, and the 30% refusal of BP measurement did not seem to affect our estimates of prevalence of high BP, as the overall crude and adjusted prevalences were similar, 27.5 versus 26.1%. An additional limitation is that we were only able to offer and perform dual bilateral BP measurements during one home visit, limiting our high BP determination to a short window even though BP may vary on repeat measurement at different time points^32^. To reduce the chance of overestimating high BP, we used the conservative clinic-based cut off of 140/90 mmHg rather than a lower threshold of 130/80 mmHg for defining high BP when assessed in the home setting^33,34^.

Our study also had several strengths. Our BP assessment was performed in a representative sample of the population (residents of a random sample of 20% of households from 22 communities across the country). In addition, household BP assessments may represent a better predictor of cardiovascular disease (CVD) risk than clinic BP assessment^35^. Finally, our sample size was relatively large, and included high proportions of persons living with HIV for whom we had very complete ART, CD4, and HIV-1 RNA data.

Our study highlights unacceptably high rates of high BP with high concomitant unawareness of high BP status by participants and low rates of BP control among those prescribed anti-hypertensive medications. We found high BP to be as common as HIV among young Black African adults in a community setting – highlighting the intersection of the two epidemics of HIV and hypertension. We demonstrated that being HIV-positive in this setting was not associated with an increased diagnosis of hypertension, in contrast to some prior reports. Unfortunately, unlike the impressive HIV viral suppression rates reported in this setting, community BP awareness and control rates were disappointingly low. Community-level BP screening initiatives are urgently needed, and among those found to have hypertension, their BP should be actively managed to reach recommended targets. Failure to diagnose hypertension and subsequently attain appropriate community level BP targets will adversely impact the health of individuals. Hypertension, a preventable or manageable risk factor for cardiovascular disease requires increased attention in sub-Saharan Africa.

### Supplementary Information

Supplementary Tables.

## Data Availability

Additional data supporting the study results are provided in the supplementary file. The de-identified survey data are available from the Botswana Combination Prevention Project Executive Committee for researchers who meet the criteria for access to confidential data. Data requests may be sent to the corresponding author or the following emails: mpretori@hsph.harvard.edu or jmakhema@bhp.org.bw.
