# Supplementary material for: Prevalence and control of hypertension in a high HIV-prevalence setting, insights from a population based study in Botswana
Source: Sci Rep. 2023 Oct 19;13:17814. doi: 10.1038/s41598-023-44499-4 (PMC10587125; doi:10.1038/s41598-023-44499-4)
Supplement: Supplementary file 1 — Supplementary Tables. [file 41598_2023_44499_MOESM1_ESM.pdf]

Full Title: Prevalence and control of hypertension in a high HIV-prevalence setting, insights from a population based study in Botswana

Mosepele MOSEPELE*[a,b,c]; Kara BENNETT[d]; Tendani GAOLATHE[a,b]; Joseph M. MAKHEMA[b], Mompati MMALANE[b], Molly PRETORIUS HOLME[c], Refeletswe LEBELONYANE[e], Omolola OMETORUWA[f], Lisa A. MILLS[g], Kathleen M. POWIS[b,c,h], Jean LEIDNER[i], Joseph N. JARVIS[b,g,j], Neo M. TAPELA[b,e,k], Tiny MASUPE[l], Lucky MOKGATLHE[m], Virginia A. TRIANT[h], Kathleen E. WIRTH, ScD[n]; Thato MOSHOMO [a], Shahin LOCKMAN, MD[b,c,f]

Supplementary Table 1: Baseline demographic and clinical characteristics of participants who agreed to have their blood pressure measured versus those who did not

		Overall (N=3,981)	BP Missing (N=1,198)	BP Available (N=2,783)	p-value[1]
**Age**	Mean (s.d.)	38.3(13.4)	38.2(13.1)	38.4(13.6)	0.672
	Min, Max	17.5,67.5	17.6,67.5	17.5,67.3	
	Median	35.9	36.4	35.8	
	16-24	755(19%)	229(19%)	526(19%)	0.26
	25-34	1118(28%)	322(27%)	796(29%)	
	35-44	878(22%)	284(24%)	594(21%)	
	45-54	598(15%)	188(16%)	410(15%)	
	55-68	632(16%)	175(15%)	457(16%)	
Gender	F	2547(64%)	705(59%)	1842(66%)	<.001
Relationship status	Missing	6	3	3	0.016
	Single, never married	3121(79%)	967(81%)	2154(77%)	
	Married	696(18%)	194(16%)	502(18%)	
	Widowed, divorced, or separated	158(4%)	34(3%)	124(4%)	
Education level	Missing	18	6	12	0.664
	Non-formal	467(12%)	143(12%)	324(12%)	
	Primary	836(21%)	254(21%)	582(21%)	
	Junior Secondary	1417(36%)	410(34%)	1007(36%)	
	Senior Secondary	658(17%)	196(16%)	462(17%)	
	Higher than Secondary	585(15%)	189(16%)	396(14%)	
Employment status	Missing	10	5	5	0.002
	Employed	1434(36%)	439(37%)	995(36%)	

	Unemployed and looking for work	1729(44%)	552(46%)	1177(42%)	
	Unemployed, but not looking for work	808(20%)	202(17%)	606(22%)	
Monthly income	Missing	21	10	11	0.002
	None	2430(61%)	736(62%)	1694(61%)	
	<$96	372(9%)	87(7%)	285(10%)	
	$96 to $477	911(23%)	272(23%)	639(23%)	
	>$477	247(6%)	93(8%)	154(6%)	
Time spent away from the community in the past 12 months	Missing	12	5	7	<.001
	None	2003(50%)	669(56%)	1334(48%)	
	<1 week	681(17%)	168(14%)	513(18%)	
	1 to 2 weeks	314(8%)	87(7%)	227(8%)	
	3 weeks to <1 month	303(8%)	96(8%)	207(7%)	
	1 to 3 months	478(12%)	125(10%)	353(13%)	
	>4 months	190(5%)	48(4%)	142(5%)	
at baseline survey: How often did you drink alcohol in the past month	Missing	508	160	348	0.009
	Never	2781(80%)	798(77%)	1983(81%)	
	Once a week	294(8%)	95(9%)	199(8%)	
	2 to 3 times a week	265(8%)	93(9%)	172(7%)	
	More than 3 times a week	133(4%)	52(5%)	81(3%)	
Number of sexual partners in past 12 months	Missing	368	101	267	0.234
	None	733(20%)	225(21%)	508(20%)	
	1 partner	1947(54%)	570(52%)	1377(55%)	
	2 or more partners	933(26%)	302(28%)	631(25%)	
Alcohol use during most recent sex act	Missing	551	159	392	0.014

	Both participant and partner	108(3%)	45(4%)	63(3%)	
	Partner only	189(6%)	48(5%)	141(6%)	
	Participant only	99(3%)	36(3%)	63(3%)	
	Neither participant or partner	3034(88%)	910(88%)	2124(89%)	
Smoking: Ever		534(13%)	171(14%)	363(13%)	0.296
Smoking: Current		357(67%)	123(72%)	234(64%)	0.087
Smoking: Ever counselled?		275(51%)	81(47%)	194(53%)	0.19
Salt intake: Ever counselled?		1426(36%)	380(32%)	1046(38%)	<.001
Weight: Ever checked in past 3 years?		2426(61%)	646(54%)	1780(64%)	<.001
Any counselling on weight control?		1260(32%)	359(30%)	901(32%)	0.134
Physical Activity: Ever counselled?		1486(37%)	417(35%)	1069(38%)	0.031
Alcohol intake: Ever counselled?		1050(26%)	334(28%)	716(26%)	0.158
Cholesterol: Ever screened?		536(13%)	210(18%)	326(12%)	<.001
Diabetes: Ever screened?		838(21%)	265(22%)	573(21%)	0.277
SBP (mmHg)	N	2784	1	2783	
	Mean (s.d.)	120.6(18)	114(.)	120.6(18.1)	
	Min, Max	68,243	114,114	68,243	
	Median	119	114	119	
DBP (mmHg)	N	2783	0	2783	
	Mean (s.d.)	80.5(12.2)	.(.)	80.5(12.2)	
	Min, Max	40,158	., .	40,158	
	Median	80	.	80	
Ever diagnosed with hypertension?		396(10%)	89(7%)	307(11%)	<.001
HTN meds: Ever		350(9%)	74(6%)	276(10%)	<.001

HTN meds: Currently		350(9%)	74(6%)	276(10%)	<.001
WHR: Females	N	1369	11	1358	
	Mean (s.d.)	0.872(0.199)	0.864(0.133)	0.872(0.199)	
	Min, Max	0.395,2.644	0.706,1.162	0.395,2.644	
	Median	0.842	0.847	0.841	
WHR: Males	N	676	6	670	
	Mean (s.d.)	0.887(0.153)	0.904(0.066)	0.887(0.153)	
	Min, Max	0.415,2.853	0.837,0.99	0.415,2.853	
	Median	0.865	0.885	0.865	
Current HIV status	Missing	21	12	9	<.001
	HIV-uninfected	2764(70%)	784(66%)	1980(71%)	
	HIV-infected	1196(30%)	402(34%)	794(29%)	
Current ARV status	ART-naive	70(6%)	36(9%)	34(4%)	0.005
	ART defaulter	16(1%)	5(1%)	11(1%)	
	On ART	1110(93%)	361(90%)	749(94%)	
Current CD4 count (cells/ul)	N	318	103	215	0.365
	Mean (s.d.)	586.7(255.1)	568(247)	595.7(258.9)	
	Min, Max	751,567	881,518	751,567	
	Median	542.5	534	544	
Energy source for cooking	Missing	75	26	49	0.8
	Charcoal/wood	2333(60%)	693(59%)	1640(60%)	
	Gas	955(24%)	282(24%)	673(25%)	
	Electricity (main)	598(15%)	190(16%)	408(15%)	
	Electricity (solar)	13(0%)	4(0%)	9(0%)	
	No cooking done	7(0%)	3(0%)	4(0%)	
Water source	Missing	77	26	51	<.001
	Piped indoors	440(11%)	136(12%)	304(11%)	
	Standpipe or tap within plot	2371(61%)	716(61%)	1655(61%)	
	Communal tap	617(16%)	145(12%)	472(17%)	
	Neighbor or relative's standpipe	293(8%)	85(7%)	208(8%)	
	Borehole	6(0%)	1(0%)	5(0%)	
	Bowser or tanker	152(4%)	72(6%)	80(3%)	

	Buy from others	21(1%)	17(1%)	4(0%)	
	River, dam, lake or pan	4(0%)		4(0%)	
Toilet facility	Missing	75	26	49	0.092
Toilet facility	Flush toilet within plot	609(16%)	213(18%)	396(14%)	
	Pit latrine within plot	2907(74%)	845(72%)	2062(75%)	
	Neighbor's flush toilet	37(1%)	7(1%)	30(1%)	
	Neighbor's pit latrine	261(7%)	77(7%)	184(7%)	
	Communal flush toilet	3(0%)	1(0%)	2(0%)	
	Communal pit latrine	3(0%)	1(0%)	2(0%)	
	Bush	86(2%)	28(2%)	58(2%)	
Flooring type	Missing	75	26	49	0.288
	Dirt or earth	182(5%)	51(4%)	131(5%)	
	Wood or plank	12(0%)	5(0%)	7(0%)	
	Tile	544(14%)	175(15%)	369(13%)	
	Cement	3162(81%)	941(80%)	2221(81%)	
	Other	6(0%)		6(0%)	
Number of people per room	N	3311	1020	2291	0.546
	Mean (s.d.)	2.122(1.557)	2.146(1.672)	2.111(1.504)	
	Min, Max	0.09,15	0.13,14	0.09,15	
	Median	1.67	1.67	1.67	
Electrical Appliances	Radio	2374(63%)	675(60%)	1699(64%)	0.01
	Television	2263(60%)	671(59%)	1592(60%)	0.659
	Landline	270(7%)	73(6%)	197(7%)	0.283
	Cellular phone	3646(96%)	1080(95%)	2566(97%)	0.043
	Computer	380(10%)	108(10%)	272(10%)	0.501
	Internet	429(11%)	97(9%)	332(13%)	<.001
	Refrigerator	1836(48%)	544(48%)	1292(49%)	0.704
Transportation	Tractor	46(6%)	7(5%)	39(7%)	0.43
	Motorcycle or scooter	27(4%)	10(7%)	17(3%)	0.021
	Donkey or cow cart	361(49%)	73(50%)	288(48%)	0.729
	Donkey or horse	374(50%)	72(49%)	302(51%)	0.755
Number of goats owned	N	2244	659	1585	0.162

	Mean (s.d.)	7.6(12.2)	7.1(11.2)	7.9(12.6)	
	Min, Max	0,200	0,79	0,200	
	Median	2	1	3	
Number of sheep owned	N	1905	578	1327	0.715
	Mean (s.d.)	1.1(4)	1.1(4.6)	1(3.6)	
	Min, Max	0,55	0,55	0,32	
	Median	0	0	0	
Number of cattle owned	N	2195	643	1552	0.117
	Mean (s.d.)	7.8(18.1)	6.9(17.1)	8.2(18.5)	
	Min, Max	0,300	0,250	0,300	
	Median	1	0	1	
Food scarcity past 4 weeks	Missing	76	27	49	0.094
	Never	2690(69%)	811(69%)	1879(69%)	
	Rarely	481(12%)	130(11%)	351(13%)	
	Sometimes	535(14%)	157(13%)	378(14%)	
	Often	199(5%)	73(6%)	126(5%)	

[1] p-value from Chi-square for categorical variables and from two-sample t-test for continuous variables.

Supplementary Table 2. Multivariable-adjusted odds ratios (OR) and 95% confidence intervals (CI) for having blood pressure measured (ref=no blood pressure data available) among N=3,981 participating in the hypertension survey of BCPP

		**OR**	**95% CI**	**p-value[a]**
Gender	Female	0.7	(0.462, 1.059)	0.09
Employment status				0.007
	Missing or Employed	0.481	(0.293, 0.790)	
	Unemployed and looking for work	0.566	(0.395, 0.811)	
	Unemployed but not looking for work	(ref)		
Missing data on employment		0.76	(0.138, 4.166)	
Income in last month				0.001
	Missing or None	1.14	(0.716, 1.817)	

	<$96	2.018	(1.389, 2.932)	
	$96 to $477	1.489	(1.095, 2.025)	
	>$477	(ref)		
Missing data on income		0.758	(0.242, 2.378)	
Nights away from the community				0.002
	Missing or None	0.692	(0.487, 0.984)	
	<1 week	1.017	(0.694, 1.491)	
	1 to 2 weeks	0.902	(0.591, 1.378)	
	3 weeks to <1 month	0.707	(0.466, 1.074)	
	1 to 3 months	0.952	(0.641, 1.414)	
	>4 months	(ref)		
Missing data on nights away from the community		0.944	(0.205, 4.357)	
Counselled on salt intake?		1.308	(1.092, 1.567)	0.004
Weight checked in past 3 years?		1.306	(1.028, 1.660)	0.03
Any counselling on alcohol consumption?		0.819	(0.683, 0.983)	0.03
Any cholesterol screening?		0.507	(0.409, 0.629)	<.0001
Ever took any HTN medication?		2.387	(1.129, 5.047)	0.02
Current HIV status				<.001
	HIV+	0.75	(0.637, 0.882)	
Missing current HIV status		0.296	(0.121, 0.724)	
Number of people per room of household		0.982	(0.935, 1.031)	0.1
Missing data on number of people per room of household		1.174	(0.942, 1.463)	
Current HIV by current on ART		0.621	(0.393, 0.982)	0.04
Female by employment status				0.05
	Employed and Female	1.488	(0.960, 2.307)	
	Unemployed and looking for work and Female	1.723	(1.122, 2.646)	

Mssing or Unemployed but not looking for work and Male	(ref)		
Weight checked in past 3 years and Female	1.331	(0.993, 1.784)	0.06
Ever took any HTN medications and Female	0.541	(0.242, 1.208)	0.13
Number of livestock owned by household (log scale) by Female	1.085	(1.009, 1.165)	0.03
Missing data on number of livestock	0.967	(0.823, 1.135)	0.68

[a]Wald χ^2 P-values
